# Effects of Tegoprazan, Potassium-Competitive Acid Blocker, on the Gastric Emptying and Postprandial Symptoms in Healthy Humans

**DOI:** 10.1007/s10620-024-08714-0

**Published:** 2024-11-18

**Authors:** Hyung Seok Lim, Hai-jeon Yoon, Hye-Kyung Jung, Ji Taek Hong, Min Young Yoo, Eui Sun Jeong

**Affiliations:** 1 Department of Internal Medicine, College of Medicine, Ewha Womans University, 1071 Anyangcheon-Ro, Yangcheon-Gu, Seoul, 07985 Republic of Korea; 2 Department of Nuclear Medicine, College of Medicine, Ewha Womans University, Seoul, Korea; 3 Department of Internal Medicine, College of Medicine, Inha University, Incheon, Korea; 4 Department of Nuclear Medicine, College of Medicine, Inha University, Incheon, Korea

**Keywords:** Potassium-competitive acid blocker, Tegoprazan, Gastric emptying, Dyspepsia

## Abstract

**Background and Aims:**

Proton pump inhibitors are potent gastric acid inhibitors. However, they may worsen symptoms such as postprandial fullness and early satiation by reducing gastric emptying (GE). This study aims to evaluate the effects of tegoprazan, a new potassium-competitive acid blocker, on GE and dyspeptic symptoms.

**Methods:**

A randomized, double-blind, placebo-controlled design was adopted for this study. Participants underwent GE tests and responded to a questionnaire regarding gastrointestinal symptoms before and after administration of tegoprazan 50 mg. GE was assessed using scintigraphy over 4 h with a standardized solid meal.

**Results:**

Thirty participants were recruited (19 men, mean age 28.2 ± 7.3 years). After medication, no significant differences were observed in gastric half emptying time (T_1/2_) and gastric retention at 4 h (GE 4 h) between the tegoprazan and the control group (114.2 ± 48.9 min vs. 93.7 ± 34.7 min, P = 0.20; 10.1 ± 12.0% vs. 4.3 ± 5.4%, P = 0.11, respectively). Furthermore, there were no statistically significant differences detected in the changes within each group between two groups (T_1/2_, 9.9 ± 52.7 min vs. − 4.7 ± 30.5 min, P = 0.36; GE 4 h, 5.2 ± 13.9% vs. − 1.3 ± 6.5%, P = 0.12). The changes in dyspeptic symptom scores after tegoprazan administration did not significantly differ from those in the control group with no correlation between symptoms and GE parameters.

**Conclusions:**

In healthy adults, the administration of tegoprazan did not show a significant impact on GE and dyspeptic symptoms, especially postprandial fullness or early satiation.

## Introduction

The stomach acts as a temporary reservoir for the meal and initiates protein digestion through acid secretion and the enzyme precursor pepsinogen. In the proximal part of the stomach, a process known as gastric accommodation occurs. This process is crucial as it facilitates food storage and mixing with gastric juice. The distal part of the stomach plays a vital role in mixing the gastric contents and propelling them through the pylorus into the duodenum. These secretory and motor functions are regulated through endocrine, paracrine, and neural pathways [1]. Proton pump inhibitors (PPIs) are widely used in the treatment of acid-related or functional gastrointestinal (GI) disorders, encompassing gastro-esophageal reflux disease (GERD), peptic ulcer diseases, and functional dyspepsia (FD). PPIs exert their effects by inhibiting the acid-dependent pepsin activity, thereby affecting hydrolytic digestion and delaying gastric emptying (GE) of solid foods [2]. In addition, PPIs reduce the secretion of gastric juice and induce variable alterations in the volume and energy density, thereby modifying the GE of liquid food in an unpredictable manner [2]. Another hypothesis is that PPI administration induces hypergastrinemia, which impairs gastric emptying [3, 4]. However, the causal relationship between increased gastrin levels and delayed gastric emptying is not straightforward and is rather conjectural [2].

However, previous studies on the effects of PPIs on GE vary in methodology, and the accuracy of these methods can influence the study outcomes, resulting in inconsistent results [2]. While some findings suggest that PPIs do not affect liquid gastric emptying, there have been several reports indicating a reduction in solid gastric emptying.

Potassium-competitive acid blockers (P-CABs) have superior properties compared with conventional PPIs, with a rapid onset of action, longer duration of effect, consistent acid suppression regardless of CYP2C19, and, most importantly, a superior acid suppression capability [5]. Among the P-CABs, vonoprazan exhibits similar acid-suppressive efficacy, regardless of being administered before or after breakfast, as it does not require acid-catalyzed activation. Notably, its potent therapeutic efficacies have been demonstrated in GERD, peptic ulcer diseases, low-dose-aspirin/non-steroidal anti-inflammatory drug-mediated gastropathy, and *Helicobacter pylori* eradication [5]. Another P-CAB, Tegoprazan [(S)-4-((5,7-difluorochroman-4-yl)oxy)-N,N,2-trimethyl-1H-benzo[d]imidazole-6-carboxamide], is a novel, potent, and highly selective inhibitor of gastric H + /K + -ATPase, which is reported to activate gastric phase III contraction of the migrating motor complex (MMC) in a canine model treated with pentagastrin. However, its actual effect on GE is not well known [5]. With the increasing prevalence of acid-related and functional GI disorders, the search for effective and well-tolerated treatments has intensified. Moreover, the emergence of P-CABs as an alternative to traditional PPIs presents a pivotal juncture for research and clinical practice.

This study was conducted to compare the effect of tegoprazan on GE capacity among healthy volunteers using standardized methods of measurement. Additionally, we aimed to investigate the impact of tegoprazan administration on dyspeptic symptoms and its correlation with gastric emptying capacity.

## Materials and Methods

### Participants and Study Design

A single-center, prospective, double-blind, placebo-controlled, randomized, parallel comparative trial was conducted between December 2021 and October 2022, involving healthy adults without GI symptoms. The study enrolled thirty adults, comprising men and women aged between 19 and 65, who did not experience dyspepsia symptoms. Specifically, for women, the first 10 days of the menstrual cycle were included in the study period to exclude the risk of pregnancy and minimize the effects of progesterone [6]. Exclusion criteria encompassed individuals who underwent major GI surgery except for appendectomy, cholecystectomy, and Cesarean section. In addition, individuals who had taken P-CABs or PPIs within the 2 weeks before screening, pregnant or lactating women, or women of childbearing age who have not consented to use appropriate contraceptive methods during the study period. Moreover, individuals who took drugs that could affect GI motility, such as prokinetics, H_2_ receptor antagonists, and anticholinergics, within the 2 weeks preceding screening were excluded. People with a history of malignant tumors within the last 5 years, or an organic disease of the stomach or duodenum previously confirmed by endoscopy, were also excluded from the participant selection criteria.

This study was conducted in accordance with the 1964 Declaration of Helsinki and its later amendments. The protocol and informed consent were approved by the institutional review board (No. 202100910001). All participants were adults who voluntarily decided to participate and had the ability to understand and fill out the informed consent form.

At the initial visit, patients were screened based on inclusion and exclusion criteria, selected, and written consent was obtained. Vital signs were collected, and physical examinations and blood, urine, and pregnancy tests (for women) were performed. Individuals who passed the selection criteria performed baseline GE scintigraphy on visit 2. After completing GE scintigraphy, participants filled out the Nepean dyspepsia index (NDI) and the self-assessment questionnaire for dyspepsia (SEQ-DYSPEPSIA) questionnaires. Participants were randomly assigned to either a tegoprazan or a control group in a 1:1 ratio in a double-blind, randomized controlled manner. After computer-generated randomization, tegoprazan (one tablet of 50 mg) or placebo was assigned and administered orally before breakfast once a day for 3 days from the day after visit 2.

The PPI-induced gastric emptying may be more directly related to the acid-suppressive effect rather than elevated gastrin levels, and this pattern may also be applicable to PCABs. A single dose of PPI does not result in maximum acid inhibition, which is achieved only when irreversible binding and de novo synthesis are balanced, following 3–5 days of treatment [7, 8]. In contrast, tegoprazan demonstrates rapid acid secretion inhibition, with saturation of the parietal cells achieved for one day [9]. This biological activity is demonstrated that the first day of 24-h intragastric pH monitoring after administration of tegoprazan 50 mg is > 4 for 76% of the time and this degree of acid suppression is virtually identical to that achieved (80%) with 14 days of administration [10, 11]. In a recent study investigating vonoprazan’s effect on GE, serum gastrin levels increased to over 500 pg/dL after 2 weeks of treatment, yet no association with gastric emptying was observed [12]. Furthermore, unlike vonoprazan, tegoprazan induces hypergastrinemia at levels similar to PPIs. These data provide the rationale for the acute 3-day study we conducted in our research to assess the effects of tegoprazan on gastric functions and symptoms, similar to previous studies [2, 13]. Following drug administration, the participants performed a GE study, questionnaires and blood tests on visit 3.

### Gastric Emptying Scintigraphy

Gastric scintigraphy with a solid meal was performed as previously reported [14]. Solid phase GE was evaluated after ingesting a standardized test meal comprising a 99mTc sulfur colloid labeled egg mixed with 74 MBq (2 mCi) of radioactivity. Prior to the test, participants fasted for at least 8 h before the test. The participants consumed the test meal consisting of one steamed egg and six gimbap (carbohydrate 6.7 g, fat 0.06 g and protein 0.57 g per one gimbap) within 10 min. This intake was equivalent to consuming a total of 285 kcal meal (73% carbohydrate, 16% protein, 10% fat). Radioactivity was measured at eight time points over a period of 4 h using a large field-of-view gamma camera (Symbia Intevo Bold, Siemens, Germany). The initial measurement was performed immediately after ingestion of the test meal (time 0). Subsequent measurements were taken every 15 min for the first hour, followed by measures at 120 min, 180 min, and 240 min. GE was analyzed using conventional techniques [14]. A region of interest was delineated around the activity observed throughout the entire stomach, as visualized in the anterior and posterior static gamma camera images (or the left anterior oblique view if acquired). The final measurement of GE was calculated based on the percentage of gastric retention at specific time points following meal ingestion [15].

### Questionnaires

As a secondary outcome, symptoms were evaluated with structured and validated questionnaires, namely the Self-Evaluation Questionnaire for Functional Dyspepsia (SEQ-DYSPEPSIA) and the NDI. The SEQ-DYSPEPSIA comprises 11 questions mainly addressing the severity and frequency of upper GI symptoms and is divided into typical FD, major FD, and other upper GI symptoms [16]. Typical FD symptoms include epigastric pain or soreness, postprandial fullness, and early satiation corresponding to the Rome IV criteria. The major FD includes typical FD symptoms and other frequent dyspeptic symptoms such as bloating, belching, and nausea. The SEQ-DYSPEPSIA demonstrated a good internal consistency (alpha = 0.770–0.905) and an acceptable test–retest reliability (intraclass correlation coefficient = 0.733–0.859) [16]. The NDI consists of 15 symptoms evaluated for frequency, intensity, degree of distress and dyspepsia-specific quality of life (QOL) evaluation tool developed by Talley et al., and the Korean version (NDI-K) was developed using translation and reverse translation methods [17]. Upper GI symptoms are evaluated based on the preceding 2 weeks. The frequency and the degree of distress are rated on a scale of five levels, while intensity is measured on a six-level scale. The QOL encompasses 25 questions rated on a five-level evaluation index. The total scores of dyspepsia-related QOL are calculated through weighted evaluation [17].

### Statistical Analysis

We calculated a minimum sample size of 10 participants in each group based on an α level of 0.05, power (1-β) of 0.9, and effect size of 3.75, dropout rate 0.1 using independent sample t-test [18].

For the primary outcome, descriptive statistics depicting the change in residual radioactivity in the stomach before and after test meal intake were presented as the mean, standard deviation, and interquartile range for each group. The difference in change of residual radioactivity in the stomach between the two groups was assessed using a two-sample t-test according to normality. Regarding the secondary outcome, the mean and standard deviation 200B of the dyspeptic symptom scores in the questionnaire were presented, and the difference in changes between the groups was analyzed using Wilcoxon's signed rank test. Statistical analyses were conducted using SPSS 25.0 (Statistical Package for the Social Science, SPSS Ins. Chicago, USA). A p-value < 0.05 was determined to be statistically significant.

## Results

### Study Populations and Demographics

Among a total of 30 participants, 15 were assigned to the tegoprazan group and the remaining 15 to the control group. The male-to-female ratios of the control and tegoprazan groups were 11:4 and 8:7, respectively. The age of the participants ranged from 19 to 48 (mean age: 28.2 ± 7.3 years). There were no differences in demographic information and baseline characteristics between the two groups (Table 1).

**Table 1:** Demographics and background characteristics of study participants

	Control group (n = 15)	Tegoprazan group (n = 15)	*P*-value*
Sex (M:F)	11: 4	8: 7	0.26
Age, mean ± SD (years),	27.7 ± 7.7	25.1 ± 4.3	0.41
Range	20–48	19–35	0.95
Height, mean ± SD (cm)	170.6 ± 7.6	170.9 ± 8.2	0.42
Body weight, mean ± SD (kg)	72.2 ± 14.8	68.6 ± 15.5	0.31
BMI, mean ± SD (kg/m^2^)	24.6 ± 3.8	24.3 ± 3.5	0.32
Baseline symptom score			
Epigastric pain or soreness	2.1 ± 0.5	2.0 ± 0.0	0.32
Early satiation or postprandial fullness	5.5 ± 2.6	4.5 ± 1.0	0.49
SEQ typical FD	7.6 ± 2.6	6.5 ± 1.0	0.43
Bloating/belching	5.3 ± 1.5	4.2 ± 0.8	0.01
Nausea/vomiting	4.4 ± 0.8	4.1 ± 0.5	0.29
NDI dyspepsia score	5.2 ± 7.4	2.6 ± 5.5	0.10
NDI total score	8.3 ± 9.4	4.4 ± 9.7	0.04

Data are presented with mean ± SD

*BMI* Body mass index; *SD* standard deviation; *FD* functional dyspepsia; *NDI* Nepean dyspepsia index

###  Effect of Tegoprazan Administration on Solid Gastric Emptying

**Fig. 1:**
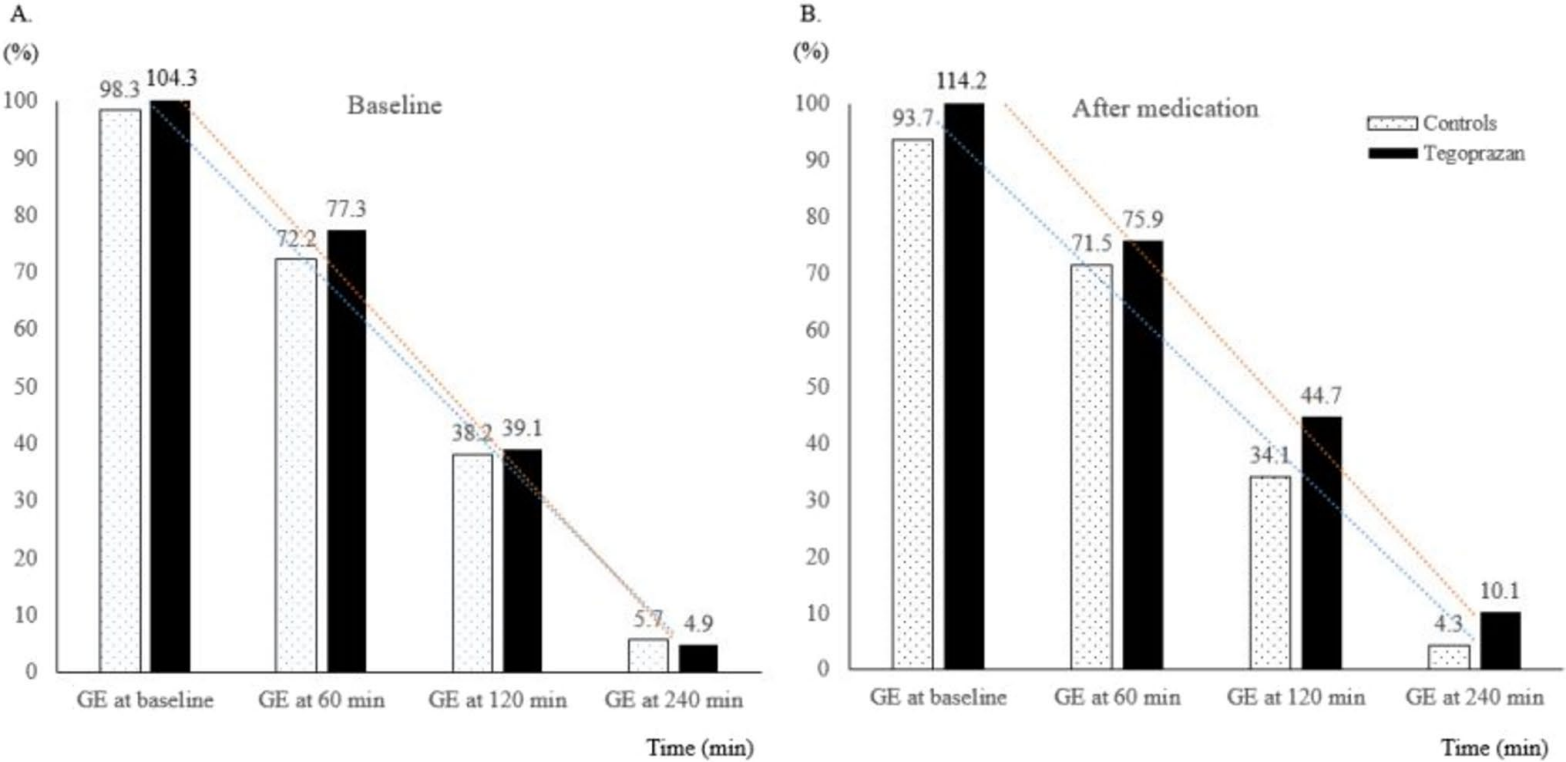
Changes of gastric emptying time for each group at baseline (**A**) and after medication (**B**)

Table 2 shows the summary of GE parameters for each group. At baseline, there was no significant difference in T_1/2_ (mean ± standard error [SE], 104.3 ± 34.8 min vs. 98.3 ± 33.0 min, *P* = 0.64) and gastric retention at 240 min (4.9 ± 6.4% vs. 5.7 ± 7.4%, *P* = 0.75) between the tegoprazan and control groups, respectively. Post-medication administration, no statistically significant differences were observed in T_1/2_ between the tegoprazan and control groups (114.2 ± 48.9 min vs. 93.7 ± 34.7 min, *P* = 0.20). Moreover, the gastric retention at 240 min in tegoprazan group was not significantly delayed compared with controls (10.1 ± 12.0% vs. 4.3 ± 5.4%, *P* = 0.11) (Figure. 1).

**Table 2:** Descriptive summary of gastric emptying parameter by group

Gastric emptying parameter	Control group (n = 15)	Tegoprazan group (n = 15)	*P*-value*
Half emptying time (min)			
Baseline	98.3 ± 33.0	104.3 ± 34.8	0.64
After medication	93.7 ± 34.7	114.2 ± 48.9	0.20
Gastric retention at 60 min (%)			
Baseline	72.2 ± 16.1	77.3 ± 18.0	0.42
After medication	71.5 ± 19.5	75.9 ± 21.7	0.57
Gastric retention at 120 min (%)			
Baseline	38.2 ± 18.5	39.1 ± 21.0	0.90
After medication	34.1 ± 21.0	44.7 ± 28.3	0.26
Gastric retention at 240 min (%)			
Baseline	5.7 ± 7.4	4.9 ± 6.4	0.75
After medication	4.3 ± 5.4	10.1 ± 12.0	0.11

Data are presented with mean ± standard error; *Comparison between two groups is analyzed with the two sample t-test

Table 3 shows the changes in GE parameters, including T_1/2_ and gastric retention (%), at various time points for each group. Across all GE parameters, the changes from baseline to post-medication administration did not display a significant difference between the groups. Moreover, no significant difference was depicted in changes in gastric retention at 240 min between the tegoprazan and control groups (5.2 ± 13.9% vs. − 1.3 ± 6.5%, *P* > 0.05).

**Table 3:** Difference between both groups in the changes in gastric emptying parameters

Change (mean ± S.E.)	Control group (n = 15)	Tegoprazan group (n = 15)	*P*-value*
Half emptying time (min)	− 4.7 ± 30.5 (*P* = 0.56)	9.9 ± 52.7 (*P* = 0.48)	0.36
Gastric retention at 60 min (%)	− 0.7 ± 18.5 (*P* = 0.89)	− 1.5 ± 22.6 (*P* = 0.81)	0.92
Gastric retention at 120 min (%)	− 4.1 ± 19.9 (*P* = 0.44)	5.5 ± 29.7 (*P* = 0.48)	0.31
Gastric retention at 240 min (%)	− 1.3 ± 6.5 (*P* = 0.44)	5.2 ± 13.9 (*P* = 0.17)	0.12

min, minutes; Data are presented with mean ± standard error

*comparison between two groups is analyzed with the two sample t-test

### Effect of Tegoprazan Administration on Upper Gastrointestinal Symptoms

The correlation analysis between upper GI symptoms and GE time (T_1/2_) showed no significance (correlation coefficient r^2^ = 0.023, *P* = 0.93). At baseline, the typical SEQ score was 6.5 ± 1.0 for the tegoprazan group and 7.6 ± 2.6 for the control group. Notably, there was no significant difference between the two groups even after drug administration (6.5 ± 1.4 vs. 7.0 ± 2.0, *P* = 0.41) (Table 4). Regarding the SEQ typical FD score scale, neither group showed significant changes (0.0 ± 0.9 in the tegoprazan group vs. − 0.6 ± 1.2 in controls, *P* = 0.14). Notably, bloating/belching scores significantly improved after placebo treatment in the control group, but no significant difference in symptom changes was observed compared to the tegoprazan group. Among the five domains of dyspepsia-related quality of life, knowledge/control domain showed significant improvement in the tegoprazan group. (Table 5).

**Table 4:** Difference in upper gastrointestinal symptom scores between treatment groups

Symptom score (mean ± S.E.)	Control group (n = 15)	Tegoprazan group(n = 15)	*P*-value^*^
Baseline			
Epigastric pain or soreness	2.1 ± 0.5	2.0 ± 0.0	0.32
Post prandial distress**	5.5 ± 2.6	4.5 ± 1.0	0.49
SEQ typical FD	7.6 ± 2.6	6.5 ± 1.0	0.43
Bloating/belching	5.3 ± 1.5	4.2 ± 0.8	0.01
Nausea/vomiting	4.4 ± 0.8	4.1 ± 0.5	0.29
NDI dyspepsia score	5.2 ± 7.4	2.6 ± 5.5	0.10
NDI total score	8.3 ± 9.4	4.4 ± 9.7	0.04
After medication			
Epigastric pain or soreness	2.1 ± 0.5	2.1 ± 0.3	0.96
Post-prandial distress	4.9 ± 2.0	4.5 ± 1.3	0.61
SEQ typical FD	7.0 ± 2.0	6.5 ± 1.4	0.41
Bloating/belching	4.3 ± 0.7	4.3 ± 0.7	1.00
Nausea/vomiting	4.2 ± 0.6	4.0 ± 0.0	0.15
NDI dyspepsia score	3.3 ± 6.2	1.5 ± 3.4	0.39
NDI total score	4.7 ± 6.8	2.5 ± 6.4	0.10

Data are presented with mean ± standard error; *comparison between two groups is analyzed with the Mann–Whitney test; **post-prandial distress includes early satiation and postprandial fullness

**Table 5:** Difference in dyspepsia-related quality of life scores between treatment groups

Difference between groups	Control group (n = 15)	Tegoprazan group (n = 15)	*P*-value^*^
Baseline			
Tension/Sleep	83.5 ± 13.4	83.6 ± 15.1	0.92
Interference with daily activities	73.8 ± 10.4	65.8 ± 13.3	0.04
Eating/Drinking	87.5 ± 12.6	82.9 ± 15.7	0.42
Knowledge/Control	72.0 ± 19.6	74.5 ± 13.9	0.95
Work/Study	78.7 ± 12.5	73.7 ± 13.2	0.41
After medication			
Tension/Sleep	84.9 ± 13.6	83.7 ± 15.1	0.80
Interference with daily activities	75.4 ± 10.2	65.8 ± 13.3	0.02
Eating/Drinking	88.4 ± 14.5	83.3 ± 16.1	0.38
Knowledge/Control	73.6 ± 20.1	74.3 ± 14.1	0.75
Work/Study	80.6 ± 12.9	73.8 ± 13.2	0.27

Data are presented with mean ± standard error; *Comparison between two groups is analyzed with the Mann–Whitney test

## Discussion

This study has shown that a therapeutic dose of tegoprazan, known for its strong suppression of gastric acid secretion, did not affect gastric solid emptying and upper GI symptoms, especially postprandial fullness or early satiation in healthy adults. P-CABs have a more potent therapeutic efficacy than PPIs [2]. Unlike PPIs, tegoprazan reversibly inhibits H + /K + -ATPase without a need for any conversion. The potency of the inhibition of acid secretion on day 1 was almost the same as that of PPIs on day 5, indicating that tegoprazan achieves maximal efficacy from the initial day of treatment. Interestingly, prior several studies had shown delayed solid GE associated with PPIs usage [2]. In addition to its potent acid inhibitory effect, tegoprazan stimulated phase III contractions of the migrating motor complex, a series of GI prokinetic contractions in the interdigestive state, in both dogs and humans [5, 19]. These prokinetic effects, including MMC stimulation, could contribute to the minimal impact of tegoprazan on solid GE.

In a systematic review conducted by Sanaka et al. [2], out of the 25 collected studies, among the 14 studies that investigated the effects of PPIs on solid gastric emptying (GE), 10 studies reported a delay in GE, while 4 studies reported no effect. Several previous studies reported that gastric acid suppression by H_2_-receptor blockers or PPIs was associated with delayed postprandial solid GE [20]. Ranitidine, which is an H_2_ receptor antagonist with cholinergic properties, famotidine which is an H_2_ receptor antagonist with minimal cholinergic effect, and PPIs, which has no cholinergic effect [21, 22], have been associated with prolongation of postprandial GE. This effect is explained by several mechanisms. The inhibition of gastric acid secretion may suppress the activation of pepsinogen, leading to a decrease in the breakdown of solid food into smaller particles, potentially delaying GE [1, 23]. Another hypothesis suggests that inhibition of gastric acid secretion by acid-suppressing agents is accompanied by a decrease in gastric fluid secretion, which, in turn, can increase the viscosity of gastric contents and potentially affect GE [21]. However, no significant differences were reported in gastric volume with rabeprazole 20 mg bid compared to controls with no correlation with solid GE [24].

Other studies have shown that exogenous hypergastrinemia stimulates GE, associated with increased motilin secretion in duodenal acidification [25]. However, a recent study has shown that a 2-week administration of vonoprazan, a newly released P-CAB in Japan, induced hypergastrinemia but did not demonstrate a significant correlation with GE [12]. While this study reported a decrease in liquid GE with vonoprazan, it is important to note that the measurement of GE was performed using liquid food instead of solid food, and the observation period was limited to post-prandial 120 min using breath tests, indicating potential limitations in its methodology. Furthermore, clinical studies evaluating administration of tegoprazan for 4–8 weeks depicted an increase in gastrin plasma concentration of approximately 1.5–2 times. However, these levels remained within the normal range of < 200 pg/mL [26]. Therefore, P-CAB induced hypergastrinemia is not considered to be directly involved in gastric motility. In this study, tegoprazan did not affect solid GE; this lack of effect may be associated with another mechanism, such as the prokinetic effect of tegoprazan. Several studies have reported that an increase in gastric pH with acid secretion inhibitors stimulates gastric phase III MMC [13]. In a canine in vivo study, intravenous administration of pentagastrin increased gastric acid secretion, which inhibited gastric phase III MMC. However, tegoprazan administration effectively and rapidly suppressed gastric acid secretion, restoring the impact of pentagastrin on GE [5]. The exact mechanism underlying the activation of phase III contractions by acid secretion inhibitors remains unclear.

FD is a common and chronic disease. While gastric acid secretion inhibitors and prokinetics are known as representative therapeutic drugs, the multifactorial pathogenesis of FD limits the efficacy of a single drug. If GE capacity is reduced, symptoms such as postprandial or early satiation may worsen, complicating FD management. In clinical settings, abnormal phase III MMC has been observed in patients with FD, and studies have reported improvements in postprandial dyspeptic symptoms associated with restoring phase III MMC [19]. The effect of tegoprazan in stimulating phase III MMC contractions may act as a compensatory mechanism for impaired GE due to acid secretion inhibition, potentially improving GI symptoms in patients with FD or GERD, further research needs to be conducted in the future to support these mechanisms. Given the current emphasis on personalized medicine and patient-centered care, our study's exploration of tegoprazan’s effects on GE and dyspeptic symptoms aligns with the evolving needs of patients and practitioners.

This prospective randomized control study has several strengths. First, to our knowledge, this is the first study to evaluate the effect of P-CAB on GE as a standardized 4-h GE protocol using a standardized solid meal. Second, symptom changes before and after treatment were assessed using two structured validated questionnaires, the SEQ-DYSPEPSIA and the NDI. On the other hand, this study also has some limitations. First, study participants were healthy young adults with a mean age of 28.3, indicating a relatively young. Elderly individuals are more likely to be prescribed medications that affect gastrointestinal motility, and since people without comorbidities were prioritized for selection, so primarily younger participants were included. And it remains unclear if similar results would arise in patients with GERD or FD. In patients with GERD or FD, tegoprazan may influence GE capacities in a different way than in healthy adults. Second, while the absence of organic disease was confirmed through the questionnaire for participation in the clinical trial, organic disease cannot be excluded entirely because evaluations such as esophagogastroduodenoscopy or abdominal-pelvic CT were not performed. Another potential limitation is the sample size. Our study was based on a parallel-group design with 15 subjects, and had sufficient statistical power to detect changes in postprandial gastric emptying. A post hoc analysis using the observed variation in gastric emptying at 4 h in this study showed that 10 participants per group would be required to demonstrate a significant effect. The previous studies have shown that another PPI, lansoprazole, does not affect gastric emptying [27]. However, the post hoc analysis suggests that, if there is a 20% (0.2) difference in the gastric emptying at 4 h, a significant effect of rabeprazole versus placebo could be demonstrated with 12 participants per treatment group. Therefore, our study cannot entirely rule out the possibility that tegoprazan may not delay gastric emptying.

In conclusion, tegoprazan 50 mg once daily did not significantly affect solid gastric emptying and did not influence dyspeptic symptoms, particularly postprandial fullness or early satiation, in healthy adults. This study provides a critical foundation for estimating the efficacy of tegoprazan use in patients with dyspepsia. Further research is needed to explore the effects of tegoprazan, especially in non-reflux dyspepsia, particularly in postprandial distress syndrome.

## Data Availability

No datasets were generated or analysed during the current study.
