# Correction: Effects of Tegoprazan, Potassium‑Competitive Acid Blocker, on the Gastric Emptying and Postprandial Symptoms in Healthy Humans

**DOI:** 10.1007/s10620-025-09019-6

**Published:** 2025-04-07

**Authors:** Hyung Seok Lim, Hai-jeon Yoon, Hye-Kyung Jung, Ji Taek Hong, Min Young Yoo, Eui Sun Jeong

**Affiliations:** 1 Department of Internal Medicine, College of Medicine, Ewha Womans University, 1071 Anyangcheon-Ro, Yangcheon-Gu, Seoul, 07985 Republic of Korea; 2 Department of Nuclear Medicine, College of Medicine, Ewha Womans University, Seoul, Korea; 3 Department of Internal Medicine, College of Medicine, Inha University, Incheon, Korea; 4 Department of Nuclear Medicine, College of Medicine, Inha University, Incheon, Korea

**Correction to: Digestive Diseases and Sciences (2025) 70:1091–1098** 10.1007/s10620-024-08714-0

The article Effects of Tegoprazan, Potassium‑Competitive Acid Blocker, on the Gastric Emptying and Postprandial Symptoms in Healthy Humans, written by Hyung Seok Lim, Hai‑jeon Yoon, Hye‑Kyung Jung, Ji Taek Hong, Min Young Yoo and Eui Sun Jeong, was originally published under exclusive license to © The Author(s), under exclusive licence to Springer Science+Business Media, LLC, part of Springer Nature 2024. As a result of the subsequent decision to publish the article under the open access model, the article’s copyright notice was changed on 19 February 2025 to © The Author(s) 2024 and the article is now distributed under a Creative Commons Attribution 4.0 International License, which permits use, sharing, adaptation, distribution and reproduction in any medium or format, as long as you give appropriate credit to the original author(s) and the source, provide a link to the Creative Commons licence, and indicate if changes were made. The images or other third party material in this article are included in the article’s Creative Commons licence, unless indicated otherwise in a credit line to the material. If material is not included in the article’s Creative Commons licence and your intended use is not permitted by statutory regulation or exceeds the permitted use, you will need to obtain permission directly from the copyright holder. To view a copy of this licence, visit http://creativecommons.org/licenses/by/4.0/.

The original article has been corrected.

